# Soil Salinity Differentiates Winter Triticale Genotypes in Physiological and Biochemical Characteristics of Seedlings and Consequently Their Yield

**DOI:** 10.3390/ijms252312971

**Published:** 2024-12-02

**Authors:** Gabriela Golebiowska-Paluch, Iwona Stawoska, Małgorzata Jelonek-Kozioł, Aleksandra Wesełucha-Birczyńska, Andrzej Kornaś

**Affiliations:** 1 Institute of Biology and Earth Sciences, University of the National Education Commission, Podchorążych 2, 30-084 Kraków, Polandandrzej.kornas@uken.krakow.pl (A.K.); 2 Faculty of Chemistry, Jagiellonian University, Gronostajowa 2, 30-387 Kraków, Poland; aleksandra.weselucha-birczynska@uj.edu.pl

**Keywords:** catalase, chlorophyll *a* fluorescence, ^13^C discrimination, peroxiredoxin, photosynthetic pigments, Raman spectroscopy, salinity stress, yielding capacity

## Abstract

The aim of this study was to test the hypothesis that both the winter triticale genotype and salinity treatment influence the photosynthesis efficiency and content of metabolites and proteins, including antioxidant enzymes, under field conditions, as well as that these parameters are correlated with yielding capacity. The research material involved four genotypes differing in their tolerance to stress in previous tests. Chlorophyll *a* fluorescence parameters and antioxidant activity were assessed in the seedlings. Specific antibodies were then used to verify the involvement of selected proteins. Simultaneously, Raman spectroscopy was employed to detect chlorophyll, carotenoids, phenolic compounds, and protein levels. The findings suggest that improved PSII performance, reduced catalase activity, increased pigment levels, and higher thioredoxin reductase abundance in the seedlings were associated with better yield potential in triticale genotypes grown under salt stress conditions. The Raman analysis revealed that salinity caused changes in the photosynthetic pigments, particularly carotenoids. The carbon isotope ratios indicate that the salinization generated different physiological stresses in the availability of water.

## 1. Introduction

Cereal fields constitute the majority of agricultural areas and, among them, winter triticale (×*Triticosecale* Wittmack) ranks second in terms of cultivated area in Poland. According to FAOSTAT data for 2022, the area harvested for triticale was close to 4 million ha worldwide, and a regular increase in its production is recorded. This laboratory-derived species combines a high protein content in grains, characteristic of common wheat (*Triticum aestivum* L.), with the ability to yield under stressful conditions after rye (*Secale cereale* L.). Most varieties currently cultivated are hexaploid triticale with genome AABBRR [1]. Triticale grains can be used as feed, but there is also growing interest in their potential use in the food industry. Crops that were infected by diseases or attacked by pests can be used to produce bioethanol and bioplastic [2].

The soil salinization considered in this work describes the degree of salt saturation or contamination when the content of dissociated salts is above 0.2%. The cause may be the lack of sufficient rainfall, excessive evaporation, lack of proper drainage, or inappropriate use of agricultural management. It is very often accompanied by high temperatures and even drought [3]. Excessive accumulation of pollutants—atmospheric acid precursors (nitrogen, sulfur, and carbon dioxide), nitrogen fertilizers, and toxic metals—can also cause soil acidification [4]. Soil salinity higher than tolerated by the species cultivated in a given area causes significant economic losses and reduced yield [5]. Currently, there are opportunities to model crop recommendations, taking into account a number of existing conditions [6,7].

Areas affected by excessive salinity are currently estimated at over 1 billion hectares, and according to analyses, this area will continue to expand [8]. The regions most affected by this problem are those located in places that force farmers to irrigate fields with low-quality groundwater. This applies mainly to arid and semi-arid areas [9]. In Poland, the area of saline soils is estimated at over 5.500 ha. It mainly concerns coastal and nearby provinces (Pomerania, West Pomerania, and Kuyavian–Pomerania) with the following soil types: rusty and luvisol, brown soils proper, acidic and leached, alluvial soils proper, brown, and black, as well as black soils proper and degraded. In these parts of the country, salinization is associated with the infiltration of waters containing salts and the rise in sea levels. Another source includes numerous industrial centers and the waste from them, which, if stored incorrectly, may influence the environment [10]. Saline areas with a high degree of industrialization are the Silesian regions with the following soil types: luvisol, brown soils proper, acidic and leached, brown alluvial soils, brown and black rendzinas, and black soils proper. Additionally, according to Pahalvi et al. [11], the source of salinization, which is caused by humans, is the excessive use of mineral fertilizers, which introduce excessive amounts of salt into the soil. The main components in the soil, the excess of which contributes to its salinization, are sodium, calcium, or magnesium cations and sulfate or chloride anions [9]. The excess of these components degrades the soil’s structure and reduces the permeability and permeability of deeper soil layers [12].

The effect of soil salinity on plants can be assessed through chlorophyll *a* fluorescence measurement, which is known to be a reliable indicator of stress-induced physiological and biochemical changes in the photosynthetic system [13,14]. Catalase (CAT) activity may reflect an increase in reactive oxygen species (ROS) under stress exposition and thus could also be an indicator of physiological status [15]. In chloroplast, a thiol-specific peroxidase (PrxQ) catalyzes the reduction in hydrogen peroxide and organic hydroperoxides, as well as plays a role in cell protection against oxidative stress by detoxifying peroxides in reaction to stress factors [16]. Raman spectroscopy allows for the simultaneous examination of various biomolecules belonging to saccharides, photosynthetic pigments, lipids, or proteins and the evaluation of molecular changes occurring in tested samples under biotic and abiotic stress factors, infections, fertilization treatments, or various agricultural applications [17,18,19,20]. It is worth underlining that this technique does not need any previous sample preparation and does not cause the destruction of the sample.

The stable carbon isotope ^13^C in nature has an overall abundance of 1.11‰, while the abundance of ^12^C is 98.89‰. During carbon acquisition and assimilation from the inorganic CO_2_ in the environment, there are different mechanisms affecting discrimination against the heavy isotope ^13^C. Isotope discrimination is influenced by stomatal conductance to CO_2_, diffusion of CO_2_ through the leaf mesophyll, as well as other thermodynamic consequences of discrimination in metabolic reactions. All these effects are quantitively overruled by the large differences between the ^13^CO_2_ discriminations of the key enzymes of primary CO_2_ fixation, namely RubisCO (ribulose-1,5-bisphosphate carboxylase/oxygenase) and PEPC (phosphoenolpyruvate) carboxylase [21]. Most photosynthesizing plant species are equipped with these two carboxylating enzymes. Plants with primary CO_2_ fixation via RubisCO have highly negative *δ*^13^C values, while plants where PEPC dominates CO_2_ fixation have less negative *δ*^13^C values due to the high and low discrimination, respectively. Some insights into biomass building and water relations can be provided by analyses of ^13^C/^12^C carbon isotope discrimination during photosynthesis [22,23,24].

As can be seen from the review of the available literature, no physiological or biochemical analyses such as those presented by us have been carried out so far for triticale seedlings and adult plants under field cultivation conditions subjected to salt stress. The aim of this work was to verify the hypothesis that both plant genotype and soil salinity affect photosynthetic efficiency, as well as the content of metabolites and proteins. For this purpose, chlorophyll *a* fluorescence and catalase activity were measured, and the abundance of thioredoxin reductase was verified using specific antibodies. In parallel, chlorophyll, carotenoids, phenolic compounds, and proteins were profiled using Raman spectroscopy. Furthermore, carbon isotope ratios in seedlings were identified, and yield capacity was assessed. The plant material consisted of model winter triticale genotypes, differentiated in tolerance to abiotic and biotic stress factors. The Hewo cultivar involved in the presented studies was used in breeding programs to develop further varieties, such as Panteon, registered by Strzelce Plant Breeding-IHAR Group Ltd., Strzelce, Poland. It is characterized by high yield regardless of the region of occurrence and soil type, very good frost resistance, and high protein content in the grain. This has a positive effect on the parameters of the produced feed. This cultivar copes well in conditions of periodic water shortage and also tolerates acidified soil complexes. Triticale Panteon is resistant to most cereal diseases, showing the greatest resistance to fusarium head blight, brown rust, and rynchosporium. In addition, this cultivar has the highest possible weight per hectoliter. The remaining triticale genotypes used in the present study included three doubled haploid (DH) lines selected from the cv. Hewo x cv. Magnat mapping population.

## 2. Results

### 2.1. Soil pH

On the day of measurements and sampling for analysis, the pH of the soil after salinization was lower (*p* = 0.0000) compared with the soil treated with only water and before any treatments (Table 1).

### 2.2. Analysis of Chlorophyll a Fluorescence

Significant effects of the seedling genotype cultivation conditions and the interaction of these factors (genotype × conditions) on the PSII quantum yield of light-adapted sample in steady-state (Fv/Fm) values were observed. Moreover, the plant genotype, as well as the treatment (cultivation conditions), also influenced the non-photochemical excitation energy quenching (NPQ) values (Table 2 and Table S1).

**Figure 1:**
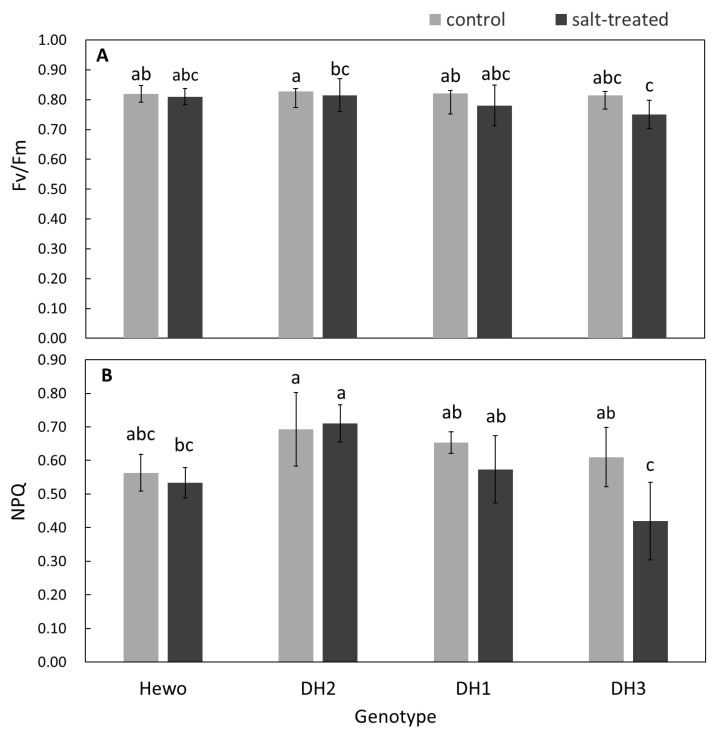
Values of chlorophyll *a* fluorescence parameters: (**A**) PSII quantum yield of light-adapted sample in steady-state—Fv/Fm recorded using portable fluorimeter and (**B**) NPQ, measured using stationary fluorimeter in field-grown winter triticale seedlings of the DH1−3 lines and the parental cultivar Hewo (HW) 96 h after salt treatment in comparison with control seedlings. The bars present average values ± SD (n = 20); the letters indicate the similarities or differences between the compared groups in terms of the value of the dependent variable according to the Kruskal–Wallis test.

In the group of seedlings exposed to salinity stress, the highest mean Fv/Fm values were observed for the cv. Hewo, while the lowest were observed for the DH3 line (Figure 1A). The maximal experimental values of the NPQ parameter were observed for the salt-treated DH2 seedlings, while minimal values were noted for salt-treated DH3 ones. Only DH3 seedlings showed an NPQ decrease after exposure to salt in comparison with the control plants of this genotype (Figure 1B).

For the control seedlings, the highest mean active reaction centers per absorption (RC/ABS) values were observed for the cv. Hewo and DH2 lines, while the lowest were observed for the DH1 and DH3 lines. The minimal experimental mean value of the potential photochemical efficiency Fv/F_0_, the ratio of variable fluorescence (1 − Vj)/Vj, and performance index PI parameters were observed for the control DH1 seedlings. The maximal relative variable fluorescence from F_0_ to Fm—V(OP) values were noted for DH2 control seedlings. In contrast, all control seedlings showed similar non-photochemical fluorescence quenching index (qN) and open reaction center fraction of PSII (qL) records. In the group of seedlings exposed to salinity stress, the highest mean RC/ABS, Fv/F0, (1 − Vj)/Vj, and PI values were observed for the cv. Hewo and DH3 lines, while the lowest were observed for the DH1 and DH2 lines. However, the maximal V(OP) values were observed in the cv. Hewo and DH2 lines. For the cv. Hewo plants, a significant increase in this value was also noted in the salt-treated plants compared with the control plants of this genotype (Table 3).

### 2.3. Antioxidative Enzymes

**Figure 2:**
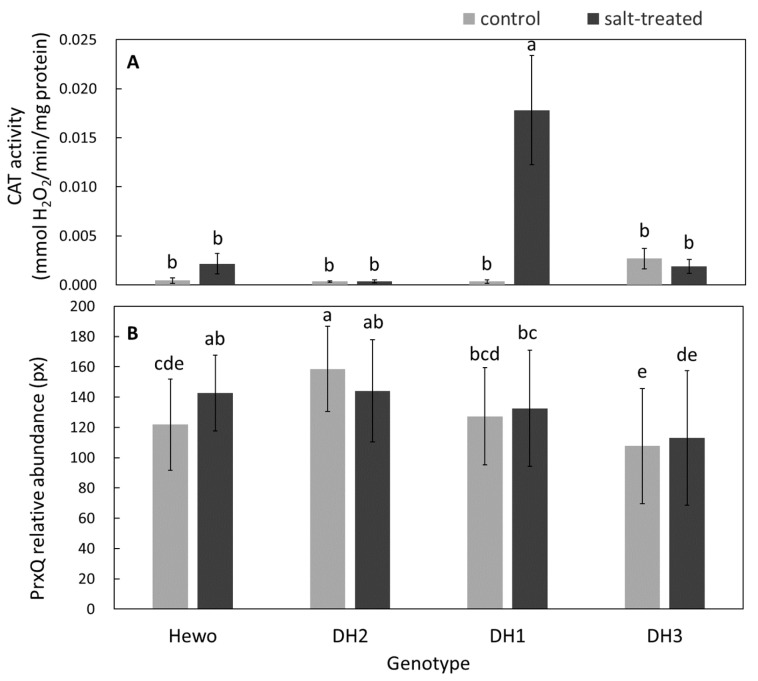
Antioxidative enzymes were assessed by (**A**) a catalase activity assay and (**B**) thiol-specific peroxidase PrxQ abundance using the immunoblotting technique in field-grown winter triticale seedlings from the DH1-3 lines and the parental cultivar Hewo (HW), 96 h after salt treatment in comparison with control seedlings. The bars represent mean values ± SD (n = 4), and the letters denote similarities or differences between the groups in terms of the dependent variable based on the Kruskal–Wallis test.

Catalase (CAT) activity level was increased only in DH1 salt-treated seedlings, both in comparison with control plants of this genotype as well as in relation to all remaining objects (Figure 2A).

In contrast, thiol-specific peroxidase (PrxQ) abundance (sample image on Figure S1) was increased only in cv. Hewo salt-treated seedlings in comparison with the control plants of this genotype and the level observed in DH2 salt-treated plants. For both control and treated DH3 seedlings, the minimum experimental values were recorded simultaneously, whereas for the DH2 control and treated plants, maximum values were observed (Figure 2B).

### 2.4. Sugar Content

**Figure 3:**
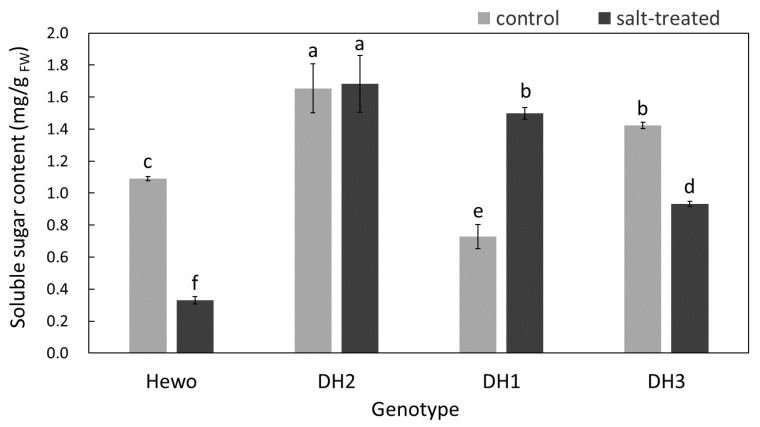
Total soluble sugar content measured spectrophotometrically in field-grown winter triticale seedlings of the DH1-3 lines and the parental cultivar Hewo (HW) 96 h after salt treatment in comparison with control seedlings. The bars present average values ± SD (n = 4); the letters indicate the similarities or differences between the compared groups in terms of the value of the dependent variable according to the Kruskal–Wallis test.

The maximal content of soluble sugars was noted in DH2 seedlings both in control and after salinization. It increased only in DH1 salt-treated seedlings in comparison with control plants of this genotype. In contrast, in plants of the remaining genotypes—cv. Hewo and DH3—it decreased under salt stress (Figure 3).

### 2.5. Pigment Content

**Figure 4:**
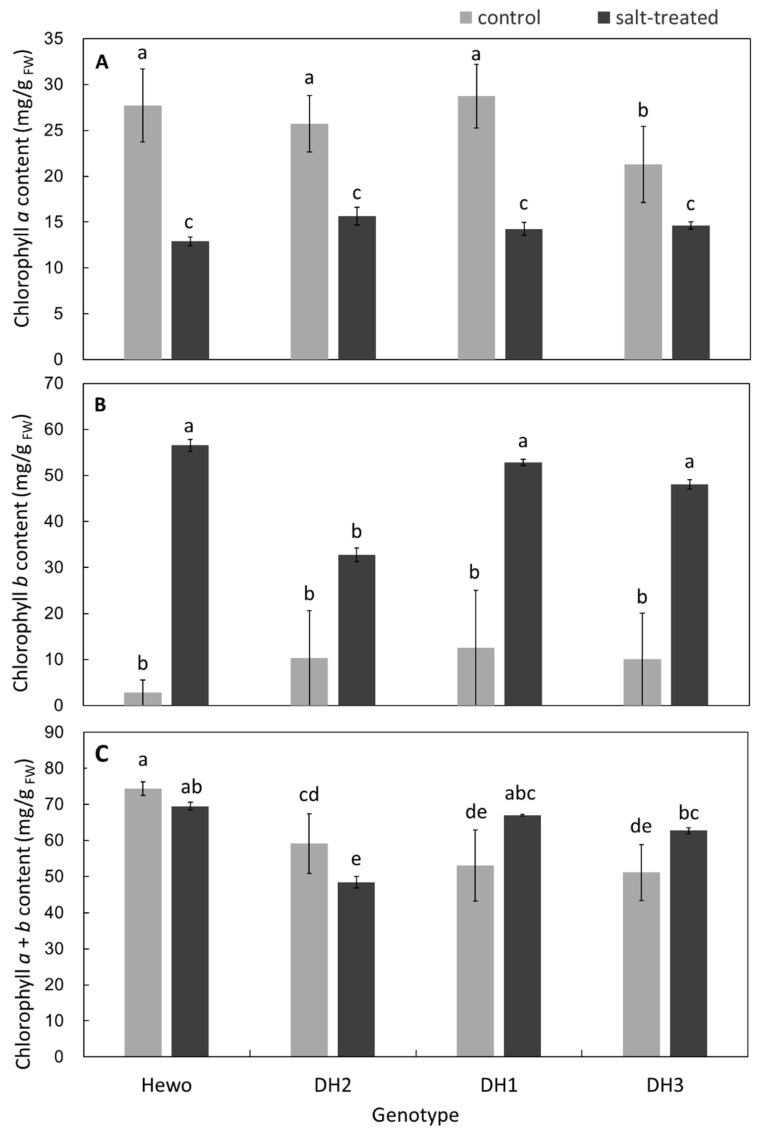
Photosynthetic pigments: (**A**) chlorophyll *a*, (**B**) chlorophyll *b*, and (**C**) chlorophyll *a* and *b* content, measured spectrophotometrically in field-grown winter triticale seedlings of the DH1–3 lines and the parental cultivar Hewo (HW) 96 h after salt treatment, in comparison with control seedlings. The bars represent mean values ± SD (n = 4), and the letters denote similarities or differences between the groups in terms of the dependent variable based on the Kruskal–Wallis test.

Chlorophyll *a* content decreased in salt-treated seedlings of all genotypes to the same level. It was also similar in the group of the control plants, with the exception of the DH3 ones, with lower content observed in these (Figure 4A). In contrast, chlorophyll *b* content was the highest in salt-treated cv. Hewo seedlings. In DH1 and DH3 seedlings, it increased after salinization to the level observed in cv. Hewo (Figure 4B). The total content of chlorophylls *a* and *b* was again the highest in both salt-treated and control cv. Hewo seedlings. In DH1 and DH3 seedlings, it increased after salinization to the level observed in cv. Hewo. In contrast, it was decreased to the minimal level observed in DH2 salt-treated plants (Figure 4C).

### 2.6. Raman Profiling

**Figure 5:**
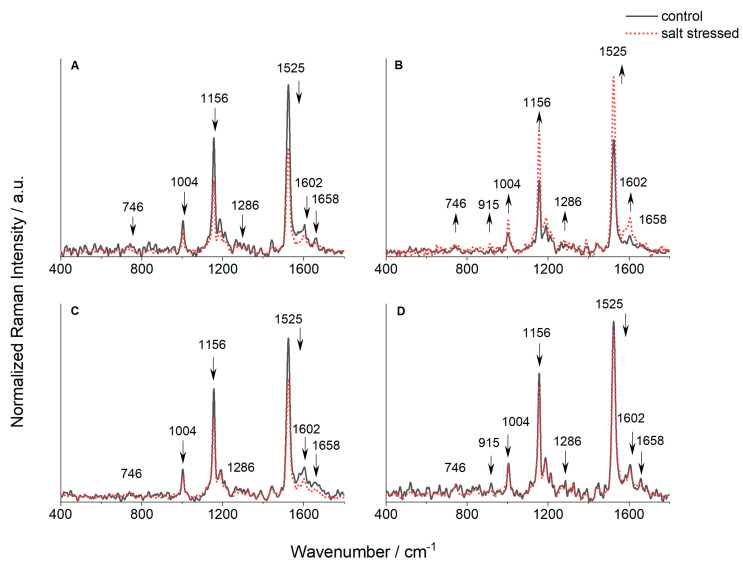
FT-Raman spectra, normalized at 1325 cm^−1^, were obtained for lyophilized leaves of seedlings grown under field conditions for the following: (**A**) DH1 line, (**B**) DH2 line, (**C**) DH3 line, and (**D**) HW cultivar. Solid black line—control leaves spectra; dotted red lines—spectra obtained for stressed plants (100 mM NaCl), 96 h after stress factor was applied. n ≥ 3.

From the averaged and normalized spectra acquired through Fourier Transform Raman measurements on lyophilized leaves of different triticale genotypes (DH1, DH2, DH3, and HW), vibrational bands corresponding to key biomolecules—such as chlorophylls, carotenoids, phenolic compounds, and proteins—were identified and characterized. The most prominent bands originated from the carotenoid triplet, appearing at 1004, 1154, and 1525 cm^−1^, as shown in Figure 5A–D [25,26]. The first band corresponds to –CH_3_ groups attached to the main chain coupled with –C-C- bonds. Bands with a maximum at 1156 and 1525 cm^−1^ are characteristic of C-C and C=C polyene chain stretching vibrations, respectively. As the position of the strongest carotenoid band depends on the number of conjugated C=C bonds, the samples likely contain nine conjugated bonds, commonly found in pigments like lutein, *β*-carotene, and others from the xanthophyll cycle [27]. Therefore, the most intense band (1525 cm^−1^) is related to the carotenoid content in the analyzed leaves.

Compared with the control, the intensity of the carotenoid triplet bands decreased after salinization in the DH1 and DH3 lines and slightly in cv. Hewo (Figure 5A,C,D). In contrast, in the DH2 line, the carotenoid triplet intensity significantly increased 96 h after salinization.

A similar trend was observed for the band at 1602 cm^−1^, characteristic of phenolic compounds. DH1 and DH3 lines, as well as the HW cultivar, exhibited reduced intensity after salinization, while the DH2 line showed an increase.

Chlorophyll-related bands appeared at 746, 1186, and 1286 cm^−1^, along with a shoulder near 1550 cm^−1^; the latter is due to the overlap of chlorophyll and phenolic compounds [28,29]. While changes in these peaks were minor after salinization, the DH2 line showed a slight increase in chlorophyll-related bands, whereas the DH1 line and cv. Hewo exhibited a slight decrease, and no change was observed in the DH3 line.

Additionally, a small decrease in the amide I band at 1658 cm^−1^ was seen in the DH1 and DH3 lines, as well as cv. Hewo following salt exposure, while the DH2 line showed a slight increase. This suggests that salt stress affects protein content, though the response varies by triticale genotype.

### 2.7. ^13^C Isotope Discrimination

There is a difference in the *δ*^13^C discrimination value in cv. Hewo: it is less negative *δ*^13^C in stressed plants (−29.82‰) compared with the control leaves (−30.04‰) (Table 4). A shift in *δ*^13^C discrimination values in seedlings leaves of the DH1-3 lines after salt treatment compared with the control was observed, although it is statistically insignificant.

### 2.8. Yielding Capacity

Only for cv. Hewo, the straw length was similar between control and salt-treated plants. The maximal values were observed for those plants, along with the DH2 and DH3 control ones. For all DH lines, straw length was reduced after exposition to salinity. In the group of plants after salinization, the highest values were observed for the cv. Hewo and DH2 lines (Table 5).

In cv. Hewo and DH1 plants, the number of kernels in the spike was similar between control and salt-treated plants and in relation to the second genotype. In contrast, the number of kernels in the spike was decreased after salinization in DH2 and DH3 plants in comparison with the control plants of these genotypes. The maximal number was observed in DH2 and DH3 control plants, while the minimal number was in DH3 salt-treated ones (Table 5).

In the cv. Hewo and DH1 lines, the weight of kernels in the spike was similar between control and salt-treated plants. In contrast, it was decreased after salinization in DH2 and DH3 plants in comparison with control plants of these genotypes. The maximal weight value was observed in DH2 control plants, while the minimal was observed in the DH1 and DH3 salt-treated ones (Table 5).

The results for the thousand kernels weight (TKW) are placed in two homogeneous statistical groups. The group with high values included DH3 control plants, as well as both control and salt-treated DH2 and cv. Hewo plants. In turn, the second group with lower values was assigned to DH1 control plants, as well as DH1 and DH3 plants exposed to salinity stress. In the case of the latter genotype, line DH3, the difference between the control and the treatment was about 10 g (Table 5).

### 2.9. Correlation Between the Values of Measured Parameters

A strong positive correlation was detected between the chlorophyll *a* fluorescence parameters Fv/Fm, NPQ, and the number and weight of kernels per spike. Moreover, the thousand kernel weight (TKW) showed a positive correlation with NPQ values. In contrast, the straw length was negatively correlated with the level of CAT activity, as well as positively correlated with several chlorophyll *a* fluorescence parameters. Moreover, chlorophyll *a* content was positively correlated with Fv/Fm values, while chlorophyll b content was negatively correlated with NPQ values. The main Raman band intensity values were positively correlated with chlorophyll *a* fluorescence parameters RC/ABS and Fv/F_0,_ as well as total chlorophyll *a* and *b* content (Table 6).

The principal compound analysis (PCA) indicated the correlation between the values of chlorophyll fluorescence parameters qN, qL, NPQ, Fv/F_0_, and V(OP) and the content of chlorophylls, carotenoids, and total soluble sugars. In addition, a correlation was demonstrated between Fv/Fm, the content of chlorophyll *a*, catalase activity, peroxiredoxin abundance, and the field yielding capacity: straw length, kernels number, and weight, as well as the thousand kernel weight (Figure S2).

## 3. Discussion

For this study, the DH lines were chosen based on multi-year tolerance assessments conducted under controlled conditions, as detailed by Gołębiowska et al. [30]. The selection followed increasing seedling susceptibility to snow mold (P index) in the following order: DH1, DH2, cv. Hewo, and DH3. Moreover, the DH lines with differing snow mold tolerance also displayed varying levels of freezing tolerance under controlled conditions, with DH1 and DH2 being tolerant and DH3 being susceptible [31]. The selected genotypes were also characterized by different levels of powdery mildew *Blumeria graminis* (DC.) Speer. tolerance in the generative phase in field cultivation [32]: cv. Hewo and DH1 plants had the lowest, and DH3 plants were intermediate, while DH2 plants had the highest mean degree of infection in 17 experiments. In our previous work [33], performed in the field on the same plants in the earlier developmental stages, the differentiation in the physiological and biochemical parameters under natural cold-hardening conditions was also observed. Lines DH1 and DH2 reached the highest values of the measured parameters after being exposed to low temperatures. On the basis of those results, we concluded that the DH1 and DH2 lines have a higher level of natural temperature occurrence tolerance than the cv. Hewo and DH3 lines.

However, under the salinity stress, the obtained results of the tolerance level are only partially consistent with the tolerance to the above abiotic and biotic stresses. After salinization, a higher content of carotenoids and chlorophyll pigments was observed in the DH2 line, namely 55% and 30% increases, compared with the control samples. In contrast, a decrease of 36% was noted for the DH1 line for carotenoids and 30% for chlorophylls, and in the DH3 line, 25% for carotenoids, while almost no changes in the selected peak intensities were observed for the cv. Hewo line. Additionally, an increase of up to 90% in phenolic compounds was found in DH2 following salinization. Carotenoids are vital components of photosynthetic antenna complexes and reaction centers, and they contribute to antioxidant protection, pigmentation, and various other functions across different plant tissues. While salinity typically hinders the growth of vegetative organs and reduces productivity, it can also enhance their color and taste [34,35]. Our study found that salt stress had a beneficial effect on the DH2 line, increasing both carotenoid and chlorophyll pigment levels. Conversely, the other lines exhibited the opposite response. Phenolic compounds play a protective role against herbivores, pathogens, and abiotic stress, suggesting that the rise in phenolic content in the DH2 line may be part of its response to salt stress.

The highest non-photochemical excitation energy quenching (NPQ) values were recorded for the plants of the DH2 line after salinization, while the lowest for the plants of the DH3 line after salt treatment. Moreover, for the measurements of fluorescence parameters using the Handy PEA device, the highest Fv/Fm, RC/ABS, Fv/F_0_, and PI values were noted for the plants of the cv. Hewo line after salinization, while the lowest was in the DH3 line. On the other hand, for plants after salinization, higher CAT activity was observed for the DH1 and DH3 lines; an inverse relationship occurred in the DH2 line and cv. Hewo plants. The smallest differences in CAT activity between control plants and those subjected to salinity stress were obtained for the DH2 line. The highest values of the relative PrxQ content (color intensity) were recorded for the leaves of cv. Hewo seedlings and the DH2 line, which were grown under salinity stress conditions. Total chlorophyll content was the highest in cv. Hewo plants after salinization and the lowest for the DH1 line after salt treatment. For the DH1 line, the lowest values of straw length were recorded for plants subjected to salinization stress. For the DH3 line subjected to salinization, the lowest values were recorded for spike length, as well as the number and weight of kernels in the spike. In turn, the number of kernel spikes per thousand kernel weight and straw length was much higher for cv. Hewo and DH2 plants than for the DH1 and DH3 lines. The conducted analyses proved a strong, significant correlation between genotype and breeding conditions (salinity) and the combination of these factors on all analyzed morphological parameters.

Based on these results, it can be assumed that, first of all, cv. Hewo, and to a lower extent, the DH2 line, has a higher level of tolerance than the DH1 and DH3 lines. Previously low temperature and snow-mould tolerant DH2 line showed higher salinity tolerance level in the present work. The accumulation of pigments may suggest changes occurring in the studied plants in order to improve the efficiency of the photosynthesis process. For cv. Hewo plants, lower values of sugar content were observed for those grown in control conditions compared with those subjected to salinization. This may suggest the better adaptation of this cultivar to the conditions of the stressor, which results in lower sugar accumulation. Seedlings of both genotypes, DH2 line and cv. Hewo, showed the highest PrxQ abundance after salt treatment. This thiol-specific peroxidase catalyzes the reduction in hydroperoxides in chloroplasts and thus may play a role in cell protection against oxidative stress by detoxifying peroxides in reaction to stress factors [16,36,37]. In turn, lower catalase activity may indicate the presence of other effective defense mechanisms, such as peroxiredoxin activity, and consequently, a lower level of reactive oxygen species in plants of the DH2 line. Similarly, other authors indicate that the level of SOD expression is related to the level of drought tolerance in triticale and, as a result, higher yield [38,39,40,41]. However, there is no reference to other publications on the effect of salt stress itself on triticale seedlings and adult plants grown in the field.

When stomatal conductance is high, discrimination of CO2 with the heavier isotope ^13^C is lower (less negative *δ*^13^C value) than for the case when diffusion via stomata is restricted. Decreasing stomatal conductance results in the lowering of the leaf intercellular CO2 level, which finally leads to an increased water use efficiency [42]. The carbon isotope ratios clearly indicate that salinization generated physiological stress of the availability of water in the plants of Hewo, probably by increasing their water use efficiency (WUE) during photosynthesis, which is manifested by lower discrimination and a more negative δ^13^C value. The carbon isotope ratios clearly indicate that the salinization generated physiological stress of the availability of water. For that reason, the plants of cultivar Hewo probably increased their WUE during photosynthesis, which is manifested by higher discrimination and a more negative *δ*^13^C value. Numerous authors point out that although several factors are expected to be involved in making this difference in *δ*^13^C, it is appropriate to relate it generally to WUE [23,43,44,45,46,47,48,49].

Interestingly, the DH2 line is more salinity-tolerant than the DH1 line, although they show similar resistance to other stresses like low temperature and snow mold. This may be related to the differential activation of gene expression under the influence of salinity in these genotypes, which may be evidenced by a different level of biochemical parameters. However, this requires further explanation at the molecular level.

Since on the day of measurements and sampling for analysis, the soil pH after salinization was lower compared with the soil treated with water only and before any treatments, the above results can also be related to the stress of the lowered soil pH. The obtained data align with those presented by other authors [50], who explain that increased salinity can cause ion exchange between Na^+^ and H^+^, resulting in a higher concentration of protons in the soil solution and consequently lowering the soil pH. Additionally, it should be considered that salinization decreases the solubility of CO_2_ from the atmosphere in water, leading to a lower CO_2_ concentration in the salt solution compared with pure water. The presence of electrolytes promotes the dissociation of H_2_CO_3_, contributing to increased acidity. This effect results in a slight reduction in pH, which we observed in our experiment.

Based on different results for the genotypes with higher yielding capacity in comparison with the genotypes with lower yield, the physiological and biochemical parameters presented in our study can be indicated for the preliminary assessment of the level of winter triticale salinity tolerance.

## 4. Materials and Methods

### 4.1. Plant Material and Experimental Design

Three winter triticale doubled haploid (DH1-3) lines were cultured in field conditions along with parental cv. ‘Hewo’. They were selected from the mapping population derived by another method [51] from an F_1_ hybrid of cv. ‘Hewo’ (Strzelce Plant Breeding-IHAR Group Ltd., Strzelce, Poland) and cv. ‘Magnat’ (DANKO Plant Breeders Ltd., Kościan, Poland), due to their differentiation in level of tolerance to abiotic and biotic factors. Performed tests included temperature and drought tolerance; thus, the above triticale genotypes were considered suitable for studying the effects of salinity on morphological and biochemical parameters. In our previous field experiment, carried out on the same plants in earlier stages of seedling development, DH1 and DH2 lines adapted more efficiently to changing temperature conditions, including cold periods and freeze–thawing cycles, in comparison with DH3 and cv. Hewo seedlings [33].

After these tests on the natural temperature fluctuations, the plants were continuously grown on the same experimental plot. The conditions of their initial cultivation were described in detail in our previous work [33]. Weather data for the entire breeding period (28 September 2022–31 August 2023) were gathered from the closest weather stations (station codes: 350190566 and 250190390; station names: Kraków–Balice and Kraków–Obserwatorium) at https://danepubliczne.imgw.pl/data/dane_pomiarowo_obserwacyjne/, accessed on 1 September 2023 and presented in previous paper [33]. At the end of the seedling tillering phase (No. 29 in scale), on 20 April 2023, half of the plants were watered with 100 mM NaCl solution at 100 mL per plant. The remaining plants were watered with 100 mL per plant and served as control plants. After 5 days, soil pH values were measured using a pH meter (Extech EC600), and chlorophyll *a* fluorescence parameters were measured in at least 20 biological replicates. Samples were taken in replicates five days after salinization for the further analysis of chlorophyll *a* fluorescence, CAT activity, and FT Raman spectroscopic measurements, and samples were frozen at −80 °C for analysis of pigments and protein content.

### 4.2. Analysis of Chlorophyll a Fluorescence

Seedling intact leaves were analyzed in 20 biological replicates for control, and salt-treated plants were analyzed separately using a Handy PEA portable fluorimeter (Hansatech Instruments, Narborough, UK), on 25 April 2023. According to Maxwell and Johnson [52], actinic light was used for chlorophyll fluorescence excitation in PSII, and the steady-state fluorescence yield (Fs) was stabilized. The maximal fluorescence yield (Fm) was measured in leaves that were dark-adapted for 20 min. The maximum quantum yield of primary photochemistry at t = 0 (Fv/Fm) was calculated, where Fv—variable fluorescence yield.

In parallel, the JIP test parameters were evaluated according to Rapacz et al. [14] using a stationary FluorCam 700 ST 664-009656 fluorometer FMS 2 (Photon Systems Instruments, 664 24 Drásov, Czech Republic) in 20 biological replicates for freshly cut leaves of the pot-transported plants.

### 4.3. Enzymatic Assays

The protein concentration was determined in 4 biological and 4 instrumental replicates according to the method of Bradford [53]. All measurements of antioxidant enzyme activity were performed in at least 4 biological and 4 instrumental replicates. For the extraction of CAT, 0.1 g of fresh plant material was homogenized in liquid nitrogen and re-suspended in cold phosphate buffer (0.1 mol·dm^−3^ KH_2_PO_4_/Na_2_HPO_4_ pH 7.5, containing 3 mmol·dm^−3^ ethylenediaminetetraacetic acid EDTA and 2% (*w*/*v*) polyvinylpolypyrrolidone) in a proportion of 3:1 v/FW. The homogenate was centrifuged at 12,000× *g* for 5 min at 4 °C, and the supernatant was used for measuring the antioxidant enzyme activity, according to Aebi [54].

The reaction mixture contained 50 mmol·dm^−3^ potassium phosphate buffer at pH = 7.0, 0.1 mmol·dm^−3^ EDTA, 0.04% (*v*/*v*) H_2_O_2_, and 20 µL of enzyme extract in a 1 mL total volume. The decomposition of H_2_O_2_ was measured at 240 nm per 1 min in a quartz cuvette. As a unit of enzyme activity, a decrease in absorbance equal to 0.0145 was assumed (consumption of 1 µM of H_2_O_2_). The extinction coefficient of H_2_O_2_ of 42.6 mol^−1^·dm^3^ cm^−1^ was used. The spectroscopic analysis was performed using an Ultrospec 2100 pro UV/Visible spectrophotometer (GE Healthcare, UK Limited, Warszawa, Poland).

### 4.4. Sugar Content

The total soluble sugar content was recorded in at least 4 biological and 4 instrumental replicates, according to spectrophotometric method described by Chow and Landhäusser [55]. For each sample, five subsamples (50 mg) were extracted three times with 5 mL of 80% ethanol by boiling the samples in a 95 °C water bath for 10 min. After each extraction, the tubes were centrifuged at 2500 rpm for 5 min, and the supernatants of extractions combined for further analysis. Sugar concentrations were determined by the phenol–sulfuric acid method without removing the aqueous ethanol solvent. Samples were mixed with 1 mL of a phenol solution followed by the rapid addition of 2.5 mL of concentrated sulfuric acid (H_2_SO_4_). After 10 min of color development in the dark and an additional 30 min of cooling in a water bath at 22 °C, absorbance was measured at wavelengths from 465 to 505 nm in 5 nm increments using an Ultrospec 2100 pro UV/Visible spectrophotometer (GE Healthcare, UK Limited, Warszawa, Poland).

### 4.5. Pigment Content

The concentration of chlorophyll *a* and *b* was measured using the method of Lichtenthaler and Wellburn [56], with appropriate modifications. Leaves weighing approximately 0.2 g were ground in concentrated ethanol in a ratio of 1:5 (*w*/*v*, fresh weight:ethanol) in 4 biological replicates. The prepared material was centrifuged at 4 °C at 14,000 rpm, and then supernatant was collected and stored in closed tubes in a cool, dark place. Then, spectrophotometric measurements of absorbance were performed in at least 4 biological and 4 instrumental replicates at a wavelength of 664 nm to assess the content of chlorophyll *a*, as well as 649 nm for the content of chlorophyll *b*, using an Ultrospec 2100 pro UV/Visible spectrophotometer (GE Healthcare, UK Limited, Warszawa, Poland). Concentrated ethanol was used as a blank. The pigment concentration was calculated using the following formulas:

$$C_{a}=13.95A_{665}-6.88A_{649}$$

$$C_{b}=24.96A_{649}-7.32A_{665}$$

### 4.6. Western Blot

Verification of the presence and relative abundance of the peroxiredoxin (PrxQ) was carried out in 4 biological replicates and 4 instrumental replicates, according to the modified protocol provided by the Bio-Rad company, for salt-treated and control plants 96 h after NaCl/H_2_O watering. First, the total soluble protein concentration was evaluated in extracts by the method of Bradford [53]. Aliquots of 5 µg protein samples were loaded in triplicates onto 12% polyacrylamide separating gels overlayed by 4% stacking gel along with Prestained Western Blotting Protein Standards (Bio-Rad, Kraków, Poland). One-dimensional denaturing SDS electrophoresis was performed in 10 mM Tris-HCl (pH = 8.0) with 0.1 mM EDTA (TE) running buffer in replicates in a Mini-PROTEAN^®^ Tetra System electrophoresis chamber (Bio-Rad, Poland) at RT for 15 min at 24 mA/gel, followed by 30 min at 40 mA/gel. After separation, the gels were used with ready-to-use Trans-Blot Turbo Mini 0.2 µm PVDF Transfer Packs #1704156 (Bio-Rad, Poland). The electrotransfer of proteins onto the membrane was performed in a Trans-Blot^®^ TurboTM Transfer System (Bio-Rad, Poland) at 25 V/1.3 A.

After 7 min, the gels were removed, and membranes were picked for immunostaining. Before taking the next steps, the top of the first protein lane was marked on the membrane by cutting off a small fragment. To control the specificity of the antibody reaction, the membranes were first briefly incubated with the Ponceau S reagent. This resulted in non-specific staining of all protein bands present on the membrane after electrotransfer, which could then be compared with the result of specific staining. In our previous work, protein spots of the same molecular weight as those presented here and reacting with the same antibody as that used here were subjected to mass spectrometric analysis, which confirmed the identification of this protein as peroxiredoxin [36]. Then, the membranes were incubated (25 mL/membrane) in Tris Buffered Saline (TBS) with 1% casein-blocking solution (Bio-Rad, Poland). After washing three times in Tris-buffered saline with 0.1% Tween^®^ 20 Detergent (TBST) for 20 min, the membranes were incubated overnight in 25 mL of the primary antibody—polyclonal against Peroxiredoxin, thioredoxin reductase PrxQ (Q9LU86, At3g26060) of expected MW 16 kDa (AS05 093, Agisera, Vännäs, Sweden) in 1:5000 dilution in TBST solution. Next, three 20 min washes in TBST were followed by 2 h of incubation in 25 mL of solution of a secondary polyclonal antibody conjugated with the detecting system, which allowed for the visualization of specifically recognized proteins (Rabbit anti-Goat IgG (H + L) Secondary Antibody, AP, Invitrogen, Warszawa, Poland). After one short wash in TBST, the membranes were incubated for 2 min in darkness in a 5 mL/membrane solution of 5-bromo-4-chloro-3-indolyl-phosphate in conjunction with Nitro Blue Tetrazolium (BCIP/NBT Color Development ready-to-use Solution, Bio-Rad). Then, the staining solution was decanted from above the membranes and allowed to dry and decolorize the background. The received images were scanned in the ChemiDoc MP System (Bio-Rad) and analyzed with Image J 1.46 r software/Lab Program ver 4.1 (Bio-Rad, Poland).

### 4.7. FT-Raman Spectroscopy

The Raman spectra of lyophilized leaves from control and salt-treated seedlings of the Hewo (HW) cultivar and the DH1-3 winter triticale lines were obtained following the methods described by Golebiowska et al. [36] and Stawoska et al. [33]. These measurements were carried out using a Thermo Scientific Nicolet NXR 9650 FT-Raman spectrometer (Madison, WI, USA) equipped with an InGaAs (indium gallium arsenide) detector and a Micro-Stage Microscope. The excitation was performed using a 1064 nm Nd:YAG laser. The experimental setup involved a laser power of 200 mW, 500 scans accumulation, and measurement in the range of 4000–300 cm^−1^. For each leaf sample (three leaves per triticale genotype, both salt-stressed and control), at least three spectra were taken. The spectra from each sample were averaged, followed by baseline correction and background subtraction using the second derivative mode with 18 points and *β*-spline connection. The spectra were then normalized at 1325 cm^−1^, a reference wavelength characteristic of pyrrole ring breathing vibrations (from chlorophyll *a* and *b*) common to all samples. Finally, analyses were conducted in the 2000–300 cm^−1^ range to identify the vibrational characteristics of specific biomolecules. All mathematical analyses were performed using Origin Pro 2020 software.

### 4.8. ^13^C Isotope Discrimination

The frozen samples were oven-dried for 24 h at 105 °C before being ground to a fine powder for isotopic analysis. Isotope ratio measurements of C were performed on a Finnigan MAT 253 Mass Spectrometer (Thermo Finnigan, Bremen, Germany) coupled with a Flash HT Elemental Analyzer in continuous flow mode. Samples were weighed in tin capsules and introduced into the combustion furnace at a temperature of 1020 °C. A small volume of oxygen was added to the system to ensure the full combustion of organic compounds and conversion into elemental gases. CO_2_ was then separated in a chromatographic column (heated to 45 °C) and transferred in a carrier gas (He) via a ConFlo IV Interface to the isotope ratio mass spectrometer. International isotope standards were used to calculate the results: USGS 40, USGS 41, and IAEA 600 [57]. Stable carbon isotope ratios of plant material were obtained by mass spectrometry of the CO_2_-released combusted samples and expressed as

δ13C=C13/C12 of sample C13/C12 of an international standard−1×103(‰)

### 4.9. Yielding Capacity

In mature plants, the straw length, number of kernels per spike, and weight of kernels per spike were recorded, and the thousand kernel weight was calculated in at least 20 replicates.

### 4.10. Statistical Analysis

Statistical significance was assessed using the Shapiro–Wilk normality test, followed by a multi-factor ANOVA for data with a normal distribution. Kendall’s Tau correlation coefficient was calculated for all parameters, covering categorical (genotype, treatment, and genotype × treatment) and quantitative (parameter values) data. The Kruskal–Wallis test was used to indicate the similarities (same letters) or differences (different letters) between groups for the dependent variable. The δ^13^C values were analyzed by Student’s *t*-test. A statistical analysis was performed with STATISTICA^®^ version 13.0 software at *p* ≤ 0.05.

## 5. Conclusions

The obtained results may suggest better adaptation to salinity stress for plants of the Hewo variety and DH2 line compared with plants of the DH1 and DH3 lines. The specific characteristics of cv. Hewo and DH2 lines that confer this adaptation include better PSII performance together with lower catalase activity, higher pigment content, and thioredoxin reductase abundance in seedlings, as well as higher field-yielding capacity after exposure to salt stress. Correlations observed between physiological and production parameters seem useful for breeding programs. A strong positive correlation was detected between the following chlorophyll *a* fluorescence parameters: Fv/Fm, NPQ, RC/ABS, Fv/F0, (1 − Vj)/Vj, and PI with the grain yield and straw length. In contrast, the straw length was negatively correlated with the level of CAT activity.

The results of our work can be used in practice for growing triticale under conditions of soil salinity stress, although further investigation into the molecular mechanisms involved in adaptation to salt stress would be advisable. Furthermore, it is advisable to repeat the entire experiment using a different concentration of NaCl as a stressor in order to further differentiate the genotypes in terms of tolerance and to verify the responses and relationships obtained.

Field testing results, including the present study, could provide methodological or interpretive limitations connected with climatic variability, as well as biotic stresses. Cv. Hewo is already included in the Strzelce Plant Breeding-IHAR Group Ltd. breeding programs (descendant winter triticale cultivar Panteon). In turn, the DH2 line is a promising genotype for further breeding steps. In further breeding, it is also worth taking into account the obtained correlations, including the significance of straw length. It should also be further clarified whether triticale genotypes growing well on acidic soils will have increased tolerance on saline soils and vice versa.

## Figures and Tables

**Table 1:** Soil pH values before and on the day of analysis (n = 20).

Treatment	Mean pH	±SD	Rank Group at *p* < 0.05
Natural condition (initial)	6.89	0.169	a
96 h after treatment with water (control)	6.87	0.182	a
96 h after treatment with 100 mM NaCl	6.55	0.297	b

**Table 2:** The level of significance of the independent categorical factors’ impact (genotype cultivation conditions and their interaction) on the physiological and biochemical (quantitative) parameters of winter triticale seedlings grown in the field. Fv/Fm—PSII quantum yield of light-adapted sample in steady-state, maximal photochemical efficiency of PSII; NPQ—non-photochemical quenching of excitation energy; qN—non-photochemical fluorescence quenching index; qL—open reaction center fraction of PSII; RC/ABS—the active reaction centers per absorption, apparent antenna size of an active PSII; Fv/Fo—potential photochemical efficiency; (1 − Vj)/Vj—the ratio of variable fluorescence at the J-step, PI—performance index; V(OP)—relative variable fluorescence from F_0_ to Fm.

Variable	Genotype	Treatment	Genotype × Treatment
Chlorophyll *a* fluorescence parameters			
Fv/Fm	*	***	***
NPQ	**	*	ns
qN	*	*	ns
qL	*	ns	ns
RC/ABS	***	ns	*
Fv/Fo	**	ns	***
(1 − Vj)/Vj	*	ns	*
PI	****	ns	ns
V(OP)	*	*****	*****
Antioxidative enzymes			
Catalase activity	*****	*****	*****
Peroxiredoxin abundance	*****	*	*
Metabolite content			
Soluble sugars	*****	**	*****
Chlorophyll *a*	*	****	****
Chlorophyll *b*	*	*	*
Chlorophyll *a* + *b*	**	ns	*
Yield			
Straw length	*****	*****	*****
Number of kernels/spike	*****	*****	*
Weight of kernels/spike	*****	*****	*****
Thousand kernels weight	*****	**	ns

* *p* < 0.05; ** *p* < 0.01; *** *p* < 0.005; **** *p* < 0.001; ***** *p* < 0.0005; ns—not significant.

**Table 3:** Values of chlorophyll *a* fluorescence parameters: qN—non-photochemical fluorescence quenching index; qL—open reaction center fraction of PSI; RC/ABS—the active reaction centers per absorption, apparent antenna size of an active PSII; Fv/F_0_—potential photochemical efficiency; (1 − Vj)/Vj—the ratio of variable fluorescence at the J-step, PI—performance index, V(OP)—relative variable fluorescence from F_0_ to Fm, measured using stationary fluorimeter in field-grown winter triticale seedlings of the DH1−3 lines and the parental cultivar Hewo (HW) 96 h after salt treatment in comparison with control seedlings. The bars present average values ± SD (n = 20); the letters indicate the similarities or differences between the compared groups in terms of the value of the dependent variable according to the Kruskal–Wallis test.

Values of Chlorophyll *a* Fluorescence Parameters			
Genotype	Control (±SD)	100 mM NaCl (±SD)	% of Control
qN			
Hewo	0.423 (±0.0167) ^a^	0.410 (±0.0153) ^ab^	95
DH2	0.480 (±0.0300) ^a^	0.483 (±0.0133) ^a^	103
DH1	0.467 (±0.0088) ^a^	0.430 (±0.0305) ^a^	88
DH3	0.440 (±0.0264) ^a^	0.343 (±0.0405) ^b^	71
qL			
Hewo	1.22 (±0.010) ^ab^	1.20 (±0.030) ^abc^	98
DH2	1.15 (±0.027) ^abc^	1.14 (±0.021) ^c^	99
DH1	1.16 (±0.017) ^abc^	1.16 (±0.037) ^abc^	100
DH3	1.19 (±0.013) ^abc^	1.24 (±0.018) ^a^	104
RC/ABS			
Hewo	0.826 (±0.0170) ^a^	0.853 (±0.0219) ^a^	103
DH2	0.829 (±0.0211) ^a^	0.642 (±0.0588) ^c^	77
DH1	0.680 (±0.0941) ^b^	0.654 (±0.0687) ^c^	96
DH3	0.790 (±0.0300) ^b^	0.818 (±0.0225) ^a^	104
Fv/F_0_			
Hewo	4668 (±107) ^ab^	4814 (±88) ^a^	103
DH2	4430 (±121) ^ab^	3392 (±285) ^d^	77
DH1	3934 (±425) ^cd^	3946 (±432) ^cd^	100
DH3	4386 (±256) ^ab^	4570 (±84) ^ab^	104
(1 − Vj)/Vj			
Hewo	0.553 (±0.0043) ^ab^	0.569 (±0.0058) ^a^	103
DH2	0.562 (±0.0073) ^ab^	0.469 (±0.0292) ^c^	83
DH1	0.500 (±0.0413) ^bc^	0.485 (±0.0404) ^c^	97
DH3	0.574 (±0.0172) ^a^	0.556 (±0.0071) ^ab^	97
PI (ABS)			
Hewo	2132 (±70) ^ab^	2356 (±92) ^a^	103
DH2	2128 (±140) ^ab^	1798 (±141) ^bc^	77
DH1	1759 (±176) ^bc^	1607 (±252) ^c^	96
DH3	2122 (±124) ^ab^	2086 (±105) ^ab^	104
V (OP)			
Hewo	0.550 (±0.0179) ^d^	0.696 (±0.0081) ^a^	127
DH2	0.630 (±0.0129) ^bc^	0.648 (±0.0184) ^ab^	103
DH1	0.585 (±0.0338) ^cd^	0.575 (±0.0222) ^cd^	98
DH3	0.587 (±0.0306) ^cd^	0.636 (±0.0132) ^bc^	108

**Table 4:** *δ*^13^C discrimination values obtained for lyophilized leaves of seedlings in field-grown winter triticale seedlings of the DH1-3 lines and the parental cultivar Hewo 96 h after salt treatment in comparison with the control seedlings (n = 4).

*δ*^13^C (‰)		
Genotype	Control (±SD)	100 mM NaCl (±SD)
Hewo	−30.04 (±0.21)	−29.82 (±0.19) *
DH2	−30.41 (±0.43)	−30.51 (±0.58)
DH1	−30.45 (±0.19)	−30.01 (±0.14)
DH3	−29.82 (±0.12)	−29.83 (±0.25)

* The asterisk indicates the differences between the compared groups: control and salt-treated seedlings of a specific genotype in terms of the value of the dependent variable according to the *t*-test at *p* < 0.05.

**Table 5:** Yield capacity of the DH1–3 lines and the parental cultivar Hewo (HW) 135 days after salt treatment in comparison with control seedlings (n = 20).

Yield Capacity		
Genotype	Control * (±SD)	100 mM NaCl * (±SD)
	Straw length (cm)	
Hewo	88.65 (±15.99) ^a^	82.66 (±13.57) ^ab^
DH2	87.63 (±16.59) ^a^	76.58 (±14.28) ^bc^
DH1	67.31 (±10.08) ^c^	55.52 (±11.07) ^d^
DH3	83.18 (±17.13) ^a^	72.79 (±20.58) ^c^
	Number of kernels per spike	
Hewo	56.88 (±16.52) ^b^	52.20 (±14.59) ^b^
DH2	68.31 (±21.15) ^a^	48.10 (±21.24) ^bc^
DH1	49.40 (±18.08) ^bc^	39.12 (±11.73) ^bc^
DH3	57.80 (±23.25) ^ab^	31.00 (±17.04) ^d^
	Weigth of kernels per spike (g)	
Hewo	2.36 (±1.02) ^b^	2.09 (±0.78) ^bc^
DH2	3.23 (±1.41) ^a^	2.23 (±1.22) ^bc^
DH1	1.64 (±0.79) ^cd^	1.22 (±0.53) ^d^
DH3	2.50 (±1.36) ^b^	0.99 (±0.64) ^d^
	Thousand kernels weight (TKW)	
Hewo	39.19 (±11.04) ^a^	39.36 (±7.33) ^a^
DH2	45.64 (±11.74) ^a^	44.54 (±11.24) ^a^
DH1	31.97 (±10.75) ^b^	30.18 (±6.50) ^b^
DH3	39.27 (±13.55) ^a^	29.15 (±9.66) ^b^

* The letters indicate the similarities or differences between the compared groups in terms of the value of the dependent variable according to the Kruskal–Wallis test.

**Table 6:** Correlation coefficients between the studied variables, according to Kendall’s Tau test. CAT—catalase activity; Chl—chlorophyll content; TKW—thousand kernels weight; Fv/Fm—PSII quantum yield of light-adapted sample in steady-state, maximal photochemical efficiency of PSII; NPQ—non-photochemical quenching of excitation energy; RC/ABS—the active reaction centers per absorption, apparent antenna size of an active PSII; Fv/Fo—potential photochemical efficiency; (1 − Vj)/Vj—the ratio of variable fluorescence at the J-step; PI—performance index.

	Chl *a*	Chl *b*	Raman Bands	Straw Length	Number of Kernels/Spike	Weight of Kernels/Spike	TKW
CAT							
Fv/Fm							
NPQ							
RC/ABS							
Fv/F_0_							
(1 − Vj)/Vj							
PI							
Chl *a* & *b*							
TKW							
Color code legend.							
R ≥ −1.00							
R ≥ 0.62							
R ≥ 0.71							
R ≥ 0.86							
not significant							

## Data Availability

All data generated or analyzed during this study are included in this published article. Moreover, the datasets used and/or analyzed during the current study are available from the corresponding author upon reasonable request.

## Supplementary Materials

The following supporting information can be downloaded at https://www.mdpi.com/article/10.3390/ijms252312971/s1.
