# Supplementary material for: Soil Salinity Differentiates Winter Triticale Genotypes in Physiological and Biochemical Characteristics of Seedlings and Consequently Their Yield
Source: Int J Mol Sci. 2024 Dec 2;25(23):12971. doi: 10.3390/ijms252312971 (PMC11641505; doi:10.3390/ijms252312971)
Supplement: Supplementary file 1 [file ijms-25-12971-s001.zip › ijms-3311900-supplementary.pdf]

International Journal of Molecular Sciences

MDPI

Table 1 Supplementary. The level of significance (*p* values) of the independent categorical factors' impact (genotype cultivation conditions and their interaction) on the physiological and biochemical (quantitative) parameters of winter triticale seedlings grown in the field. Fv/Fm - PSII quantum yield of light-adapted sample in steady-state, maximal photochemical efficiency of PSII; NPQ - non-photochemical quenching of excitation energy; qN - non-photochemical fluorescence quenching index; qL - open reaction center fraction of PSII; RC/ABS = the active reaction centres per absorption, apparent antenna size of an active PSII; Fv/Fo -potential photochemical efficiency; (1-Vj)/Vj - the ratio of variable fluorescence at the J-step, PI - performance index and V(OP) - relative variable fluorescence from F_0 to Fm.

Variable	Genotype	Treatment	Genotype × Treatment
**Chlorophyll *a* fluorescence parameters**			
Fv/Fm	0.0289	0.0012	0.0021
NPQ	0.0054	0.0479	0.1878
qN	0.0130	0.0484	0.2407
qL	0.0274	0.8038	0.4318
RC/ABS	0.0042	0.4732	0.0149
Fv/Fo	0.0051	0.8947	0.0032
(1-Vj)/Vj	0.0282	0.3099	0.0133
PI	0.0003	0.5330	0.1987
V(OP)	0.0321	0.0001	0.0000
**Antioxidative enzymes**			
Catalase activity	0.0000	0.0000	0.0000
Peroxiredoxin abundance	0.0000	0.0341	0.0405
**Metabolite content**			
Soluble sugars	0.0000	0.0098	0.0000
Chlorophyll *a*	0.0482	0.0009	0.0009
Chlorophyll *b*	0.0423	0.0428	0.0288
Chlorophyll *a+b*	0.0092	0.2345	0.0322
**Yield**			
Straw length	0.0000	0.0000	0.0000
Number of kernels/spike	0.0001	0.0000	0.02561
Weight of kernels/spike	0.0000	0.0000	0.0000
Thousand kernels weight	0.0000	0.0084	0.1658

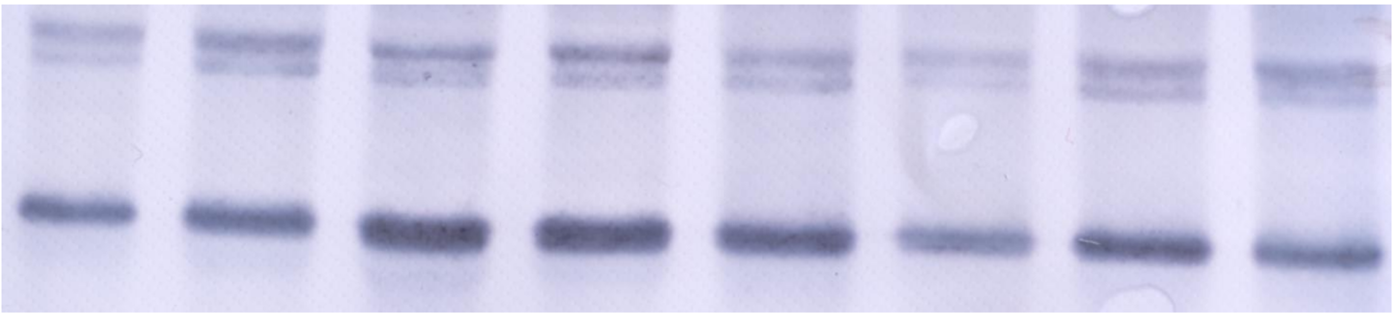

Figure 1 Supplementary. Image of an example membrane after electrotransfer of proteins after separation in a polyacrylamide gel and subsequent immunostaining with an antibody directed against the peroxiredoxin PrxQ.

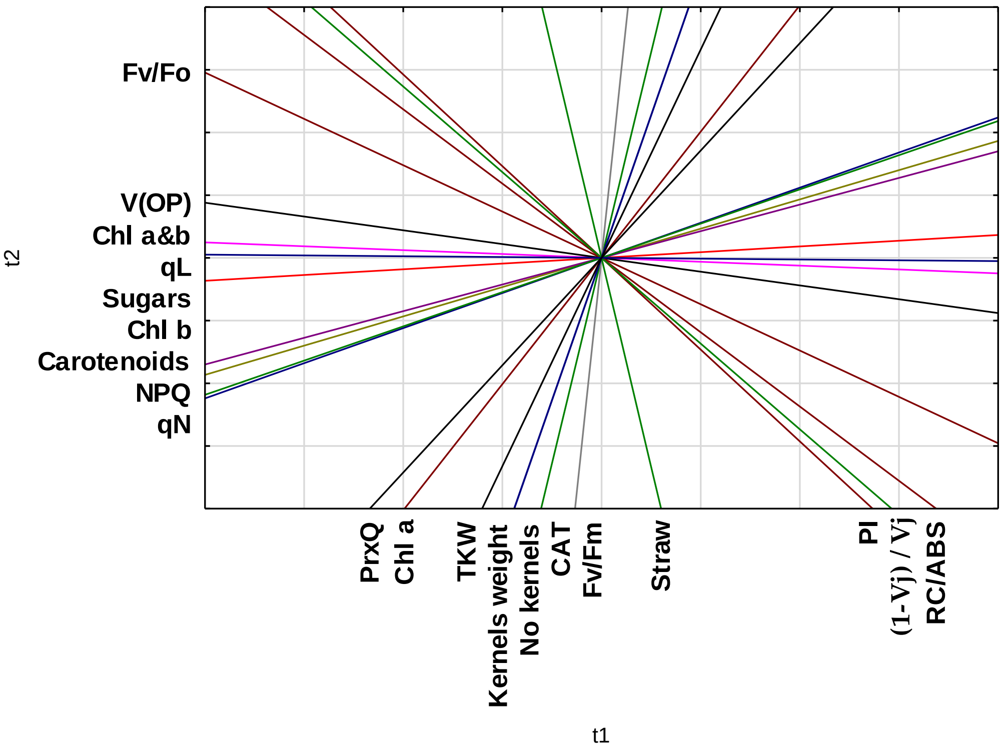

Figure 2 Supplementary. Principal Component Analysis (PCA) of the data obtained for: CAT - catalase activity; Chl - chlorophyll content; TKW - thousand kernels weight; Fv/Fm - PSII quantum yield of light-adapted sample in steady-state, maximal photochemical efficiency of PSII; NPQ - non-photochemical quenching of excitation energy; qN - non-photochemical fluorescence quenching index; qL - open reaction center fraction of PSII; RC/ABS - the active reaction centres per absorption, apparent antenna size of an active PSII; Fv/Fo -potential photochemical efficiency; (1-Vj)/Vj - the ratio of variable fluorescence at the J-step, PI - performance index; V(OP) - relative variable fluorescence from F_0 to Fm as well as yielding capacity: straw length, number of kernels per spike, weight of kernels per spike and the thousand kernel weight.
